# Supplementary material for: Oculomotor inhibition precedes temporally expected auditory targets
Source: Nat Commun. 2020 Jul 14;11:3524. doi: 10.1038/s41467-020-17158-9 (PMC7360783; doi:10.1038/s41467-020-17158-9)
Supplement: Supplementary file 2 — Reporting Summary [file 41467_2020_17158_MOESM2_ESM.pdf]

nature research

Corresponding author(s): Shlomit Yuval-Greenberg

Last updated by author(s): Apr 30, 2020

Reporting Summary

Nature Research wishes to improve the reproducibility of the work that we publish. This form provides structure for consistency and transparency in reporting. For further information on Nature Research policies, see Authors & Referees and the Editorial Policy Checklist .

Statistics

For all statistical analyses, confirm that the following items are present in the figure legend, table legend, main text, or Methods section.

n/a	Confirmed	
☐	☒	The exact sample size (n) for each experimental group/condition, given as a discrete number and unit of measurement
☐	☒	A statement on whether measurements were taken from distinct samples or whether the same sample was measured repeatedly
☐	☒	The statistical test(s) used AND whether they are one- or two-sided<br>*Only common tests should be described solely by name; describe more complex techniques in the Methods section.*
☐	☒	A description of all covariates tested
☐	☒	A description of any assumptions or corrections, such as tests of normality and adjustment for multiple comparisons
☐	☒	A full description of the statistical parameters including central tendency (e.g. means) or other basic estimates (e.g. regression coefficient) AND variation (e.g. standard deviation) or associated estimates of uncertainty (e.g. confidence intervals)
☐	☒	For null hypothesis testing, the test statistic (e.g. F, t, r) with confidence intervals, effect sizes, degrees of freedom and P value noted<br>*Give P values as exact values whenever suitable.*
☒	☐	For Bayesian analysis, information on the choice of priors and Markov chain Monte Carlo settings
☒	☐	For hierarchical and complex designs, identification of the appropriate level for tests and full reporting of outcomes
☐	☒	Estimates of effect sizes (e.g. Cohen's d, Pearson's r), indicating how they were calculated

Our web collection on statistics for biologists contains articles on many of the points above.

Software and code

Policy information about availability of computer code

Data collection	Data was collected with Matlab version 2014a
Data analysis	Data analysis was conducted with Matlab (version 2016a) and IBM SPSS (version 20)

For manuscripts utilizing custom algorithms or software that are central to the research but not yet described in published literature, software must be made available to editors/reviewers. We strongly encourage code deposition in a community repository (e.g. GitHub). See the Nature Research guidelines for submitting code & software for further information.

Data

Policy information about availability of data

All manuscripts must include a data availability statement. This statement should provide the following information, where applicable:

- Accession codes, unique identifiers, or web links for publicly available datasets
- A list of figures that have associated raw data
- A description of any restrictions on data availability

All data and codes are publicly available through OSF repository at https://osf.io/X7RSD/

Field-specific reporting

Please select the one below that is the best fit for your research. If you are not sure, read the appropriate sections before making your selection.

☐ Life sciences ☒ Behavioural & social sciences ☐ Ecological, evolutionary & environmental sciences

For a reference copy of the document with all sections, see nature.com/documents/nr-reporting-summary-flat.pdf

Behavioural & social sciences study design

All studies must disclose on these points even when the disclosure is negative.

Study description	Quantitative within-subjects experimental design.
Research sample	20 Tel-Aviv University students (14 females; Mean age 22.9 ±2.7).
Sampling strategy	Twenty-one students of Tel-Aviv University participated in the experiment for course credit or monetary compensation. One participant was discarded from all analysis due to failure to comply with the task. Consequently, eye tracking and behavioral analysis were based on a total of 20 participants.<br><br>Power analysis stimulation<br>To determine the required number of participants that will lead to power of 80% using a two-tailed criterion of .05, we conducted a simulation based on data of our previous study (Amit et al. 2019) (N=20). Data-sets were iteratively sampled (without replacement) to create sample sizes of ≥5. For each sample size, resampling was based on 10,000 iterations. We conducted a 2x5 repeated measures ANOVA on the data-set produced by each iteration, using the same factors as in the current experiment, and extracted the p value for Predictability (predictable/ unpredictable). For each sample size, we then calculated the null rejection proportion (i.e., power) out of all iterations. A sample size of 12 participants led to this result (1-β=.86), confirming that a cohort of 20 participants would be large enough to achieve reliable results with these effect sizes.<br><br>experiment 2:<br>Twenty-two students of Tel Aviv University participated in Experiment 2 for course credit. Two participants were excluded from the experiment due to ceiling performance on the task (more than 2 blocks with 100% accuracy). Consequently, eye tracking and behavioral analysis were based on a total of 20 participants (13 females; Mean age 24.9 ±4.37). All participants reported normal (uncorrected) vision and audition and no history of neurological disorders. All were naïve to the purpose of this study. The ethical committees of Tel Aviv University and the School of Psychological Sciences approved the study. All participants signed an informed consent.
Data collection	Behavioral data was recorded on a desktop computer. Eye-tracking data was collected using a remote infrared video-oculographic system (Eyelink 1000 Plus; SR Research, Canada) and stored on a separate computer. The participant sat alone in the experiment room while the experimenter monitored the experiment from outside. The experimenter was unaware to the experimental conditions.
Timing	November 2017 to December 2019
Data exclusions	One participant was excluded from analysis due to failure to comply with the task in experiment 1. two participants were excluded due to ceiling effects in two or more blocks in experiment 2.
Non-participation	No participant dropped out or declined participation.
Randomization	The study did not contain experimental groups as each task involved a within-subjects design.<br>experiment 1: blocking order (5 fixed followed by 5 random, or the opposite) was counter balanced between participants. The trials order in the random blocks were randomized. The specific order of the 5 consecutive fixed blocks was also randomized.<br><br>experiment 2: blocking order (3 predictable followed by 3 unpredictable, or the opposite) was counter balanced between participants. The trials order was randomized within each block.

Reporting for specific materials, systems and methods

We require information from authors about some types of materials, experimental systems and methods used in many studies. Here, indicate whether each material, system or method listed is relevant to your study. If you are not sure if a list item applies to your research, read the appropriate section before selecting a response.

Materials & experimental systems

n/a	Involved in the study
☒	☐ Antibodies
☒	☐ Eukaryotic cell lines
☒	☐ Palaeontology
☒	☐ Animals and other organisms
☐	☒ Human research participants
☒	☐ Clinical data

Methods

n/a	Involved in the study
☒	☐ ChIP-seq
☒	☐ Flow cytometry
☒	☐ MRI-based neuroimaging

Human research participants

Policy information about studies involving human research participants

Population characteristics	See above
Recruitment	Participants were recruited via the Tel-Aviv University research recruitment system (SONA).
Ethics oversight	The ethical committees of Tel Aviv University and the School of Psychological Sciences approved the study

Note that full information on the approval of the study protocol must also be provided in the manuscript.
